# Enhanced activations in the dorsal inferior frontal gyrus specifying the *who*, *when*, and *what* for successful building of sentence structures in a new language

**DOI:** 10.1038/s41598-023-50896-6

**Published:** 2024-01-02

**Authors:** Keita Umejima, Suzanne Flynn, Kuniyoshi L. Sakai

**Affiliations:** 1 Department of Basic Science, Graduate School of Arts and Sciences, The University of Tokyo, 3-8-1 Komaba, Meguro-Ku, Tokyo, 153-8902 Japan; 2 Department of Linguistics and Philosophy, Massachusetts Institute of Technology, 77 Massachusetts Avenue, 32-D808, Cambridge, MA 02139 USA

**Keywords:** Language, Cortex

## Abstract

It has been argued that the principles constraining first language acquisition also constrain second language acquisition; however, neuroscientific evidence for this is scant, and even less for third and subsequent languages. We conducted fMRI experiments to evaluate this claim by focusing on the building of complex sentence structures in Kazakh, a new language for participants having acquired at least two languages. The participants performed grammaticality judgment and subject-verb matching tasks with spoken sentences. We divided the participants into two groups based on the performance levels attained in one of the experimental tasks: High in Group I and Low in Group II. A direct comparison of the two groups, which examined those participants *who* parsed the structures, indicated significantly stronger activations for Group I in the dorsal left inferior frontal gyrus (L. IFG). Focusing on Group I, we tested the contrast between the initial and final phases in our testing, which examined *when* the structures were parsed, as well as the contrast which examined *what* structures were parsed. These analyses further demonstrated focal activations in the dorsal L. IFG alone. Among the individual participants, stronger activation in the dorsal L. IFG, measured during the sentence presentations, predicted higher accuracy rates and shorter response times for executing the tasks that followed. These results cannot be explained by task difficulty or memory loads, and they, instead, indicate a critical and consistent role of the dorsal L. IFG during the initial to intermediate stages of grammar acquisition in a new target language. Such functional specificity of the dorsal L. IFG provides neuroscientific evidence consistent with the claims made by the Cumulative-Enhancement model in investigating language acquisition beyond target second and third languages.

## Introduction

The Cumulative-Enhancement model (CEM) is hypothesized to account for how multiple languages are acquired; in this model, knowledge of any previously acquired languages can facilitate subsequent language acquisition^1–4^. The CEM coheres with the claim that the principles of the biological endowments that constrain the first language (L1) acquisition process also constrain the second language (L2) bilingual acquisition process^5^. In a recent functional magnetic resonance imaging (fMRI) study^6^, we obtained neuroscientific support for this model, in that the *same* syntax-related brain regions were activated for both multilinguals [L1: Japanese; L2: English; third language (L3): typically Spanish] and bilinguals [L1: Japanese; L2: English], while acquiring sentence constructions in a new subsequent language Kazakh, i.e., in an L3 for bilinguals and in a fourth language (L4) for multilinguals. Moreover, both syntax-related and domain-general brain networks were more enhanced for multilinguals than for bilinguals. Direct comparisons between the multilinguals and bilinguals showed significantly enhanced activations for the multilinguals in the *ventral* left inferior frontal gyrus (L. IFG) and right lingual gyrus (R. LG). In addition, activations of the multilinguals in the bilateral frontal and temporal regions, including the lateral premotor cortex (LPMC) and superior/middle temporal gyri (STG/MTG), were *maintained* at a higher level than the initial level during new, subsequent grammar conditions, while activations of the bilinguals in the basal ganglia/thalamus and cerebellum *returned* to the initial level at the start of each condition. While the above regions were identified as the neural substrates for multilingualism, previous studies investigating L1 and L2 acquisition have shown that the *dorsal* L. IFG is commonly recruited for syntactic processing in L1/L2^7–9^; the dorsal L. IFG is identified as the “grammar center”^10^. It should be noted that activations in the dorsal L. IFG were eliminated by the most stringent group comparisons of multilinguals versus bilinguals in the above mentioned study. On the other hand, it has been suggested that the L. IFG and L. STG/MTG form the core language regions^11,12^. In the present study, we hypothesized that the most critical syntactic processes involved in the acquisition of the grammars of the L3, L4, …, L*n* should continuously involve the same core region of the dorsal L. IFG, consistent with the CEM accounts for language acquisition. Regarding other theories and hypotheses, see our previous paper^6^. In the present paper, we aim to specify which of the cortical regions (especially dorsal L. IFG, ventral L. IFG, or L. STG/MTG) reflect the building of sentence structures in L3/L4s.

The present study is a sequel to our previous Kazakh experiments. We further elucidate the neural processes involved in successfully acquiring construction-dependent grammatical features. Both Kazakh and Japanese are agglutinative languages with a modifier-head (i.e., head-final) word order, and with a subject-object-verb (SOV) word order for declarative sentences^13^; the word orders of Kazakh sentences thus generally match those of the Japanese sentences. However, it is interesting to note that the participants in our previous and present studies reported no knowledge concerning the match in word orders; the participants were not informed about these linguistic facts during the experiments. On the other hand, subject-verb (SV) agreement (i.e., *verb suffix* in agreement with the person and number of the subject) is absent in Japanese^14^, but SV agreement is present in Kazakh, similar to English and Spanish. The participants were not informed of this syntactic difference either.

In order to understand the acquisition process described above, we used three step-wise Grammar conditions in our *previous* study to gradually familiarize participants with the syntactic structures in Kazakh: G1, G2 (after acquiring G1), and G3 (after acquiring G1 and G2) [see Supplementary Table S4 of Umejima et al. (2021) for sentence examples under G1-G3]. Those participants who could not reach criteria for each condition did not proceed to subsequent steps or levels. Under the G1 condition, we presented a *conjoined* sentence with *al* (“*and*”), consisting of [[N_1_ V_1_] *al* [N_2_ V_2_]]. We examined whether the SV agreement could be acquired for each of [N_1_ V_1_] and [N_2_ V_2_] structures (the same set of indices defines an SV pair). Under the G2 condition, we presented a *nested* sentence made up of two simple sentences with *dep* (“*that*”). We examined the construction of [N_1_ [N_2_ V_2_] *dep* V_1_], where SV agreement is applied to each of the [N_1_ V_1_] and [N_2_ V_2_] pairs, just as in G1. Under the G3 condition, we presented a sentence involving a *relative* clause with *kezde* (“*when*” or a locative form of “*time*”). We examined the construction of [[N_2_ V_2_] *kezde* N_1_ V_1_] or [N_1_ V_1_, [N_2_ V_2_] *kezde*]. In Kazakh, the suffix on an adjectival participle (V_2_ in this case) is always *fixed* regardless of the person and number of the corresponding subject (N_2_). In other words, SV agreement holds for the [N_1_ V_1_] pair for the main clause, but not for the [N_2_ V_2_] pair for the relative clause under the G3 condition. The participants, who were highly proficient and successful in passing the G1-G3 conditions in the previous study, proceeded onto the next G4 condition described here in the *present* study (see Supplementary Table S1).

Under the G4 condition, we tested sentences that consisted of a main clause and a subordinate relative clause, including nouns as *objects* (basically marked with the suffix -*di*/-*dï* in Kazakh). Each of the stimulus sentences in the G4 condition had either *adamdï* (an accusative form of *man*) or *adam* (a nominative form of *man*). For example, an adjectival participle with *adamdï* builds the structure of “N_1_ [N V_2_ *adamdï*] V_1_” (English example: “*We* (N_1_) *recognized* (V_1_) [*a man who knew* (V_2_) *John* (N)]”). An N without an index represents an *object* hereafter. With *adam*, for example, the structure of “[N_2_ V_2_ *adam*] N V_1_” is constructed (English example: “[*A man whom John* (N_2_) *knew* (V_2_)] *recognized* (V_1_) *us* (N)”). Just as in the G3 condition, SV agreement is mandatory for the [N_1_ V_1_] pair in the main clause, but not for the [N_2_ V_2_] pair in the relative clause.

**Figure 1:**
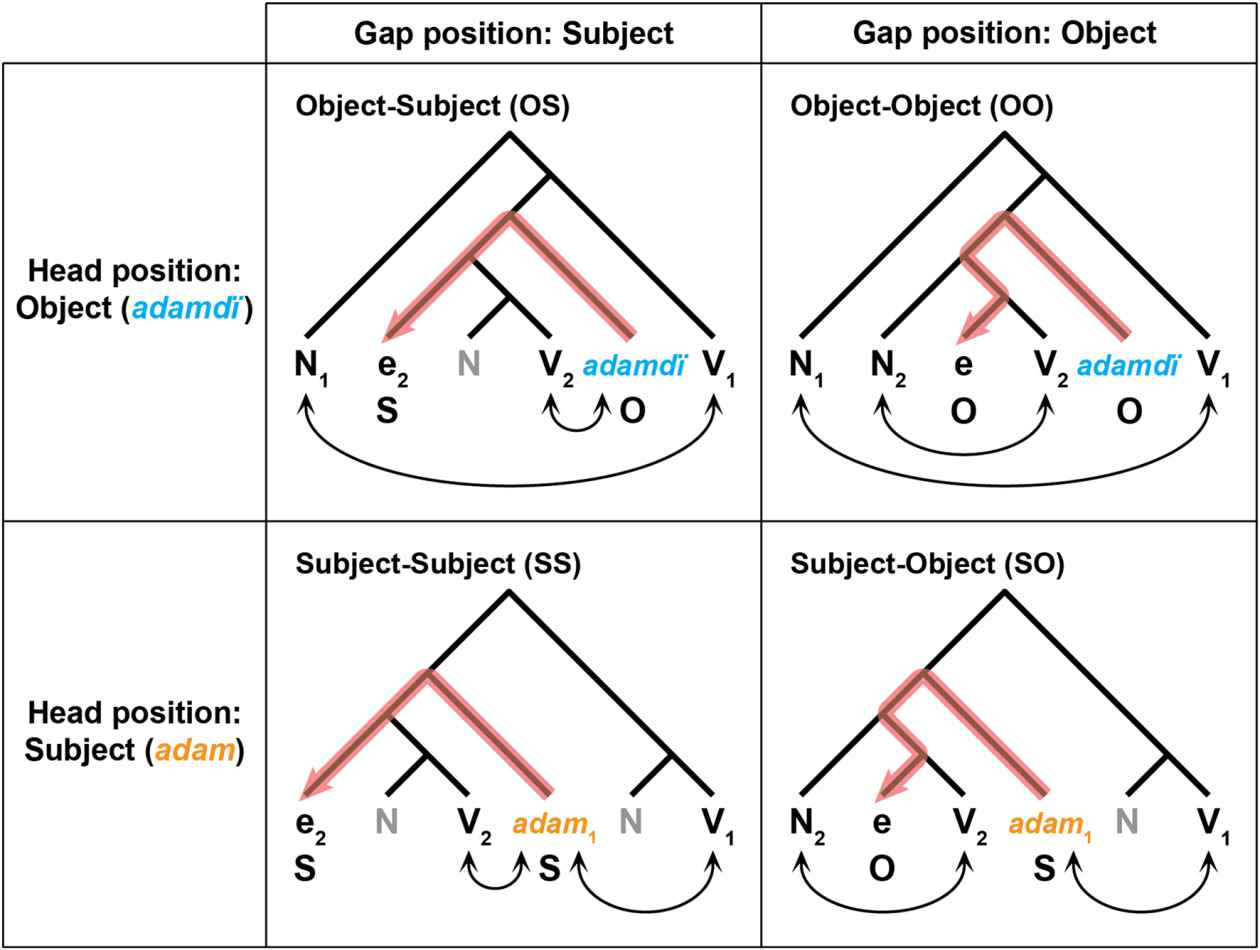
Syntactic structures of Kazakh sentences with a relative clause. We presented Kazakh sentences in one of four *construct conditions*: OS, OO, SO, and SS, which are shown here in a two-by-two format. In the relative clause (a bracket [ ] for each sentence example in Table 1), the *gap* is indicated by an empty category (e), which is not pronounced but corresponds to a *head* (*adamdï* or *adam* in these examples) in the main clause. The noun *adamdï* (in blue) is the accusative form of “*man*,” and *adam* (in orange) is in the nominative case. For each panel, a binary-branching tree structure is shown, and each red bending arrow indicates the syntactic relationship between the head and gap. For example, the “Object-Subject (OS)” construction represents the *object* at the head position (the *start* of an arrow), and the *subject* at the gap position (the *end* of an arrow). In each sentence, the same indices are attached to the corresponding subject/noun or pronoun (N) and predicate/verb (V), where the indices 1 and 2 denote the *main* and *relative* clauses, respectively. An N (shown in gray) without an index is always an object. Bidirectional arrows below the nouns and verbs denote subject-verb (SV) pairs.

For each sentence used in the G4 condition, its syntactic structure is primarily determined by a two-by-two factorial design (Fig. 1): the *head* position (either Object or Subject) in the main clause, and the *gap* position (either Subject or Object) in the relative clause. In total, four types of sentence structures were presented to the participants in randomized order, which in turn made the G4 condition more demanding than the G1-G3 conditions. With regards to the CEM, it is of great interest concerning whether the common computational system, underlying the acquisition of any language specific grammar, can be shown to be critically involved under such demanding grammatical conditions as those exemplified in the G4 condition.

In English, a *head-initial* language, an object relative clause with the head “*the man*” and the gap indicated by an empty category (e), such as (i) “*the man* [*whom John knew* e],” has the meaning of “*John knew the man*.” Brain imaging studies using English sentences with relative clauses have indicated that such *object* relatives carry increased loads for grammatical processing, i.e., higher *parsing* loads, than *subject* relatives, like (ii) “*the man* [*who* e *knew John*]”^15,16^. This increased syntactic load for the object relatives has been explained in terms of the surface structure “distance” between the head and the gap, which are both structurally and linearly farther apart in object relatives [see (i)] than in subject relatives [see (ii)]. In *head-final* languages of both Kazakh and Japanese, the distance between the head and gap becomes structurally farther apart (i.e., the gap being more deeply embedded), although linearly closer, in *object* relatives (gap position: *Object*) than it is in *subject* relatives (gap position: *Subject*); compare the red zigzagging arrows with the straight ones in Fig. 1. Example sentences with *object* relative clauses are shown as (3) and (5) in Table 1; those with *subject* relative clauses are shown as (1) and (7). The structural account for the higher syntactic load required for *object* relative clauses has been experimentally confirmed for Japanese^17^.

**Table 1:** Sentence structures under four construct conditions.

Sentence construction		Sentence example
Object-Subject (OS)		N_1_ [e_2_ N V_2_] *adamdï* V_1_
	(1)	*Biz* [*Johndï biletin*] *adamdï tanïdïq.* “*Watasitati-wa* [*John-wo yoku sitteita*] *hito-dato wakatta.*” “*We recognized a man* [*who knew John well*]*.*”
	(2)	*Biz [*Johndï ****bildi***] ***adamdï**** tanïdïq.*
Object-Object (OO)		N_1_ [N_2_ e V_2_] *adamdï* V_1_
	(3)	*Biz* [*John biletin*] *adamdï tanïdïq.* “*Watasitati-wa* [*John-ga yoku sitteita*] *hito-dato wakatta.*” “*We recognized a man* [*whom John knew well*]*.*”
	(4)	*Biz [***John biletin***] *adamdï ****tanïdï***.
Subject-Object (SO)		[N_2_ e V_2_] *adam*_1_ N V_1_
	(5)	[*John biletin*] *adam bizdi tanïdï.* “[*John-ga yoku sitteita*] *hito-wa watasitati-dato wakatta.*” “*A man* [*whom John knew well*] *recognized us.*”
	(6)	*[***John bildi***] *adam bizdi tanïdï.*
Subject-Subject (SS)		[e_2_ N V_2_] *adam*_1_ N V_1_
	(7)	[*Johndï biletin*] *adam bizdi tanïdï.* “[*John-wo yoku sitteita*] *hito-wa watasitati-dato wakatta.*” “*A man* [*who knew John well*] *recognized us.*”
	(8)	*[*Johndï biletin*] ***adam**** bizdi ****tanïdïŋïz***.

For each sentence construction, its syntactic structure (a bracket [ ] here represents a relative clause) is shown with an example of a grammatical sentence in Kazakh; its Japanese and English translations are also indicated, as well as an ungrammatical (*) example in Kazakh. In Kazakh, but not in Japanese, the verb (V_1_) in the main clause always agrees with the subject (N_1_) in person and number, while the verb (V_2_) in the relative clause is always an *adjectival participle* (see Supplementary Table S3) without SV agreement. Participants acquired such grammatical knowledge through G1-G3 trials (see the *Introduction*) without any explicit instruction. Words in boldface in the ungrammatical examples denote errors in SV agreement.

The structural distance between *adam* and the main verb (V_1_) as defined by an underlying tree structure, as well as the linear distance in the surface structure of the sentence, is greater than that between *adamdï* and V_1_. Therefore, the former (head position: *Subject*) is hypothesized to involve a higher syntactic load than the latter (head position: *Object*; see Fig. 1). Example sentences with *adamdï* are shown as (1) and (3) in Table 1; those with *adam* are shown as (5) and (7). Combining these loads together, the Subject-Object [SO; see (5)] construction with *adam* (head position: *Subject*) and an *object* relative clause (gap position: *Object*) presents the learner with potentially the *highest* syntactic load among the four constructions investigated. In contrast, the Object-Subject [OS; see (1)] construction with *adamdï* (head position: *Object*) and a *subject* relative clause (gap position: *Subject*) presents the learner with the *lowest* syntactic load. If the OS and SO constructions (see the main diagonal of the two-by-two matrix in Fig. 1) are accurately distinguished from the other constructions, then we can reasonably assume that the learner accumulated linguistic knowledge regarding the head and gap positions.

It should be noted that non-linguistic factors other than these syntactic loads, may affect task difficulty, along with constraints on short-term memory loads (including “working memory”). If so, this could reduce the accuracy rates and increase the response times (RTs) as well. It is well-known that the number of “distractors” (non-targets) influences the processing of a serial search^18^. The Subject-Subject [SS; see (7) in Table 1] construction was the most difficult to cope with regarding the tasks themselves, because there were *two* direct objects (the nouns shown in gray in Fig. 1), which were distractors in the tasks that involved the correct identification of *subject*-verb pairs (see below). In contrast, the Object-Object [OO; see (3)] construction was the easiest to cope with as there was *no* such distractor. The OS and SO constructions, in which there was *one* distractor, presented a potentially intermediate level of difficulty between the SS and OO constructions for the participants.

With respect to the relationship between L1/L2 acquisition and consequent brain activations, we had suggested earlier the possibility that “cortical activations increase initially at the onset of acquisition, followed by the maintenance of the activations and then [followed by] a fall in activations during consolidation of linguistic competence”^10^. These multiphase changes are associated with the initial, intermediate, and final stages of grammar acquisition, respectively. Multiphase changes might occur rapidly, because we observed dynamic changes in the activations for multilinguals during the time course throughout the G1-G3 conditions^6^. In the present study, we focused on the initial to intermediate stages of grammar/language acquisition, in which cortical activations should increase, for learners acquiring new syntactic knowledge in the G4 condition. Recall that this condition utilizes sentence stimuli that consisted of OS, OO, SO, and SS sentence constructions (denoted hereafter as *construct conditions*), completely mixed as in a natural language acquisition setting.

### Experimental design

In the present study, we essentially followed the design of our previous study^6^. In the design slightly modified here, we alternated between eight *demo* and eight *task* trials, in order that linguistic knowledge acquired during the demo trials would be tested in the subsequent task trials. We did not provide any explicit information about syntactic structures or rules of the grammar in Kazakh, but instead presented visual signs (either + or −) during the demo trials, where each sign indicated the status of a sentence: its grammaticality and SV correspondence. For each sentence with main and relative clause structures, three nouns and two verbs were presented, controlling for length of the sentence and number of syllables (nouns: 1–3 syllables, verbs: 2–4 syllables; see Supplementary Methods, *The Kazakh vocabulary used in this study*). With regards to the words used in the G4 condition, the participants were already familiar with these words from the previous G1-G3 experimental trials.

Before the magnetic resonance (MR) scanning was initiated, the participants were given an instruction sheet (written in Japanese) stating that, “The following examples are English translations of sentences that you will hear. There are four sentence types; each will be presented one at a time. Please note that there are objects in the sentence structure. In all the sentences, the third-person nouns *he*, *John*, *Dan*, or *man* represent different persons [thus avoiding ambiguous coreference between nouns in a sentence, but without affecting the syntactic structures].

- Example 1: The man, whom you understood, knew John,
- Example 2: The man, who knew Dan, recognized him,
- Example 3: We understood the man, whom John knew, and
- Example 4: Dan recognized the man, who knew you”.

**Figure 2:**
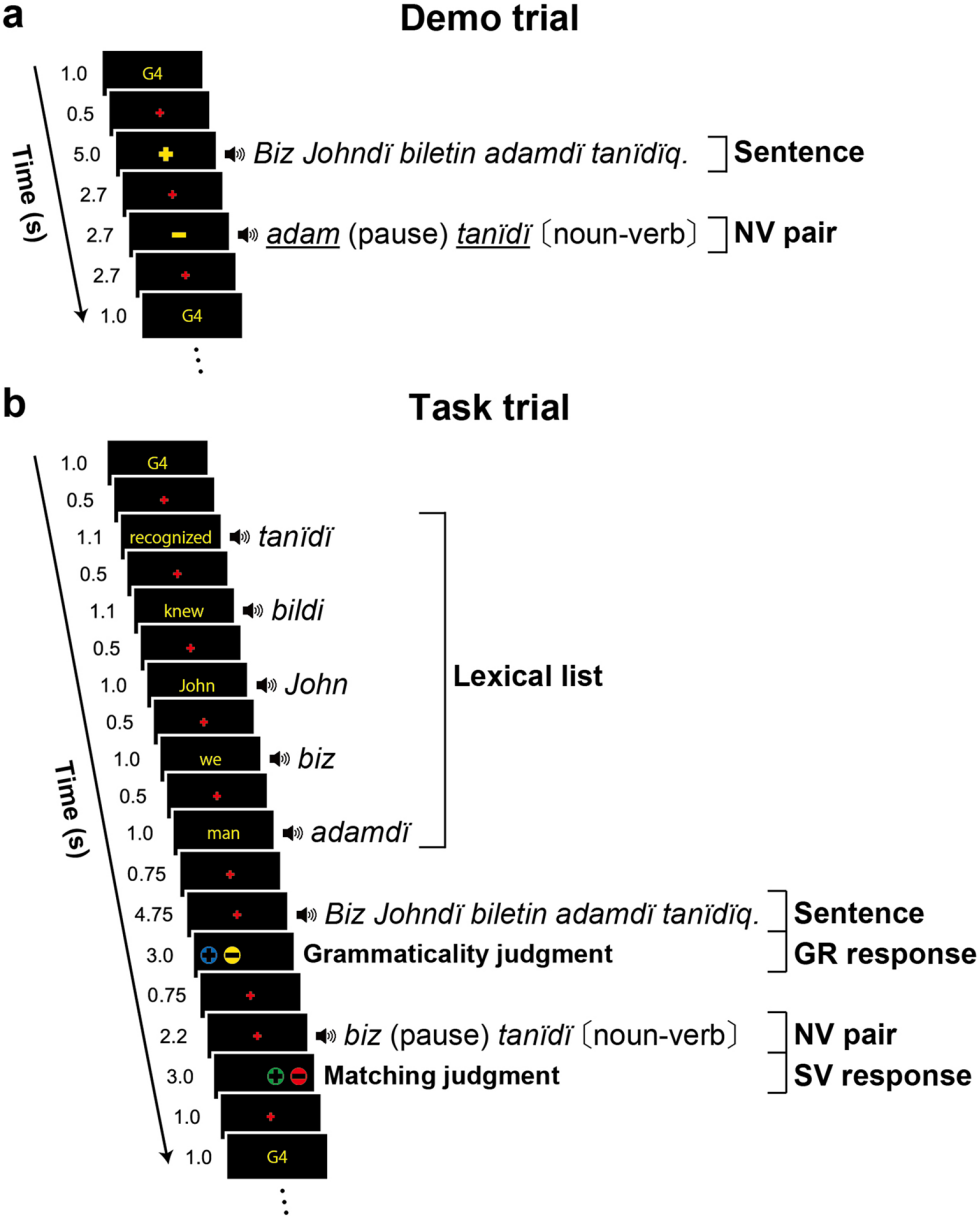
Temporal events in a demo or task trial. (**a**) In the *demo* trials, a Kazakh sentence [either grammatical or ungrammatical] was presented auditorily, followed by an NV pair [either matched or mismatched with the two SV pairs in the sentence structure; underlined words denote such a mismatch]. In each NV pair, the noun (or pronoun) was presented always without a suffix for the accusative case (e.g., *adam* for *adamdï*), and the verb was presented always with a third-person singular suffix in the simple past tense. The + / − sign presented simultaneously with a sentence indicated its grammaticality/ungrammaticality, and the + / − sign with an NV pair indicated match/mismatch (see above). The sentence shown in the figure means “*We recognized a man who knew John.*” (**b**) In the *task* trials, five Kazakh words (“Lexical list” in the figure) were presented auditorily; individual words translated into English were visually presented. This Lexical list was followed by a sentence using all five words from the Lexical list. The participants chose a + / − button in a grammaticality task (GR task). An NV pair was then presented to the participants asking them to judge the correctness of matching (see above). Here again, the participants chose a + / − button in a subject-verb task (SV task). In the activation analyses, we focused on the temporal events of the Sentence and NV pair in the task trials alone.

In the *demo* trials, each participant using headphones heard a sentence (“Sentence” capitalized here), and then a noun–verb pair (“NV pair”) extracted from the Sentence of the same trial (Fig. 2a). The noun in an NV pair was optionally a direct object in the Sentence; the noun and verb in an NV pair were presented without any inflectional suffixes. For each stimulus Sentence and NV pair, a visual sign (either + or −) was simultaneously presented on the video goggles used by each participant. The + / − sign that appeared on the goggle screen and was associated with each Sentence indicated whether the sentence was grammatical (+) or ungrammatical (−); an ungrammatical sentence always included an error in the verb suffix. The + / − sign associated with each NV pair indicated whether the pair matched (+) or did not match (−) the SV pairs in the sentence structure.

In the *task* trials, both + and − signs were presented (Fig. 2b), and the participants chose one for the Sentence, then chose another one for the NV pair (see above). These tasks have been named the *grammaticality* task (GR task) and the *subject-verb* task (SV task), respectively. While the GR task required participants to make a grammatical judgment about the Sentence, the SV task required participants to judge whether the NV pair was matched with one of the two SV pairs in the sentence structure or not, where the participants should identify an SV pair in each of the main and relative clauses. The SV task further required a syntactic analysis of the abstract empty category (see Fig. 1).

We observed a bimodal distribution for the accuracy rates in the SV task with the transition point at 60% between the two peaks under each of the OS and SO conditions (see Supplementary Figure S1). On the basis of these results, we set criteria for this experiment, such that the participants had to reach accuracy rates higher than 60% in the SV task for both the OS and SO conditions (see the above explanation for the main diagonal in Fig. 1). We separated the participants into two groups: Group I, consisting of those who had reached the criterial level, and Group II, consisting of those who did not. These two groups were formed from all of right-handed participants who had reached G4 from the previous experiment of G1-G3. This division between the two groups is no longer based on bilinguals versus multilinguals (see Supplementary Table S1), but on proficiency levels in the new language.

During the presentation of the Sentence to the participants, the processes at the lexical level involved discrimination of the nominative form of the nouns (e.g., *adam* and *John*) from the accusative form (e.g., *adamdï* and *Johndï*), as well as discrimination of the verb suffixes necessary for SV agreement. Syntactic processes were also critically involved in constructing phrase-level structures where the construction also involved the integration of both phonological and semantic information. During the presentation of the NV pair, in contrast, identification of an SV pair was required in each of the main and relative clauses. Focusing on the fMRI activations during either the Sentence or NV pair event, common and specific syntactic processes should be revealed.

According to the CEM hypothesis, if learners had more experience with their L2/L3s, higher proficiency levels would be evident in the L4 in the overall group effects, overriding individual differences. The participants with less experience in their L2/L3s would eventually become as proficient in the L4 as those participants who had sufficient experience in their L2/L3s. However, with inevitable differences in the length and/or depth of exposure to the L2/L3s, we would expect marked differences in the performances and activations in the L4. Likewise, different proficiency levels in the L4 between Groups I and II (the “*who*”) may also reflect differences in exposure to the L2/L3s as well, consistent with the CEM. Among the multi-stages involved in the acquisition of a new grammar, it is also necessary to clarify whether the initial, intermediate, and final stages (the “*when*”) are relevant for the CEM. By comparing the associated brain activations between Groups I and II, as well as between the *initial* and *intermediate* stages (i.e., initial and final phases, respectively, in our testing), we hypothesize that enhanced activations will be observed in the most crucial region among the syntax-related networks^19,20^.

## Results

### Overall proficiency improvement in Kazakh

There were large differences among the participants with respect to improving their proficiency levels in Kazakh; this made the number of task blocks for the participants variable, depending on how well they performed on the tasks (see the *Tasks* section). We divided the task blocks for each participant into four phases as equally as possible. If there were five blocks, for example, the four phases consisted of 1, 1, 1, and 2 blocks, with more blocks for the latter phases. We then averaged the accuracy rates for each *quarter* among all the participants (combining Groups I and II).

We evaluated language proficiency levels in the L2 and L3 using the *Listening Comprehension* sub-test of the Avant STAMP 4S (Standards-based Measurement of Proficiency—4 Skills; Avant Assessment, Eugene, OR, USA), as the scores of 1–9 [Novice (1–3), Intermediate (4–6), and Advanced (7–9)] (Supplementary Table S1). With respect to the fourth quarter, i.e., the final phase in our testing, Avant scores in the L2 and L3 were significantly correlated with accuracy rates in the GR task (Spearman’s correlation test, *r*_s_ = 0.53, *p* = 0.02; Supplementary Fig. S2). This result directly supports the CEM hypothesis (see the *Introduction*), regardless of large individual differences in proficiency levels, in that the more proficient the bilinguals and multilinguals were in their L2/L3s, the higher their performance became in their L4.

**Figure 3:**
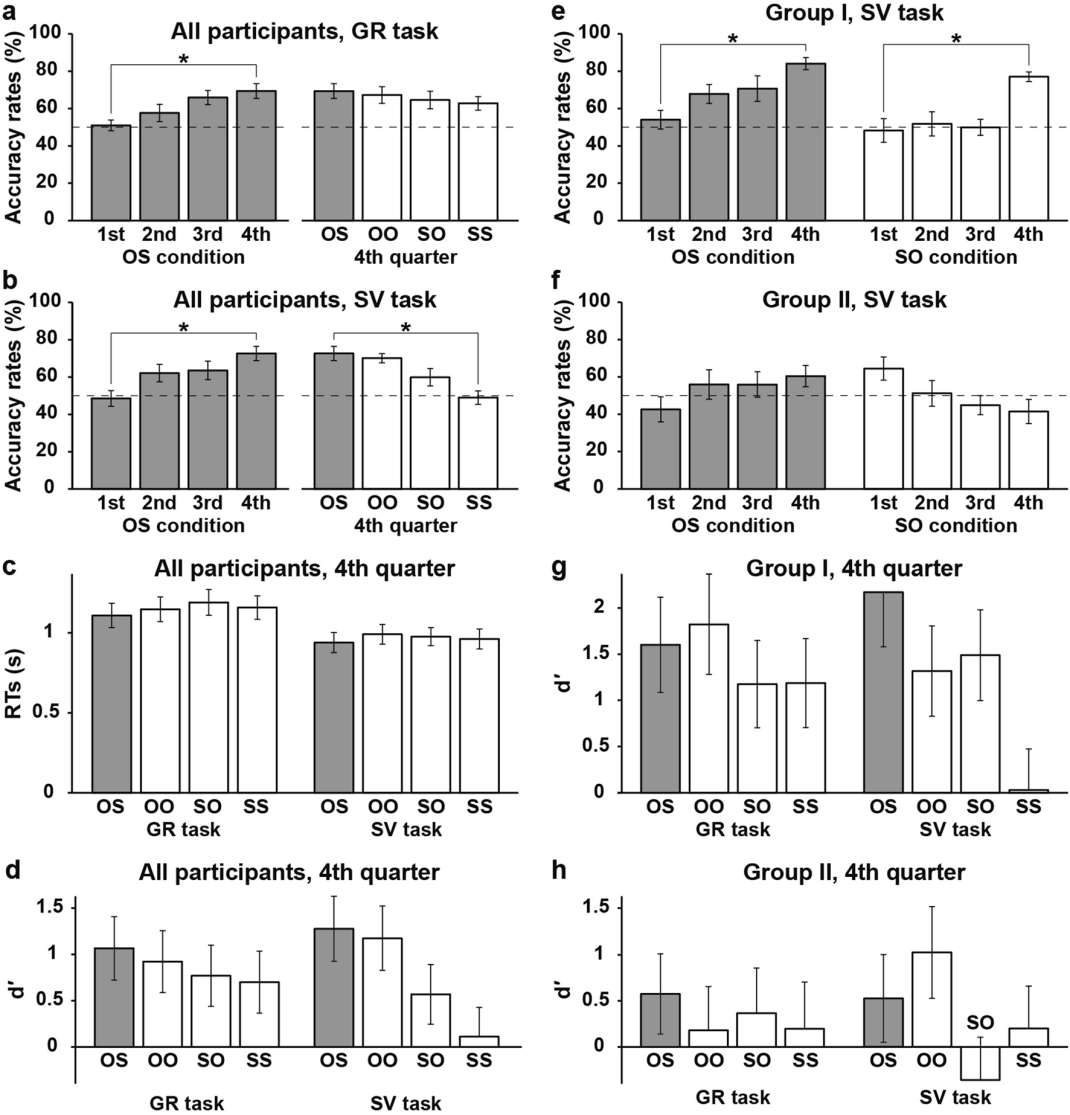
Proficiency improvement in Kazakh. (**a**) Accuracy rates in the GR task for all participants. The left panel shows the rates under the OS condition during each quarter (1st, 2nd, 3rd, and 4th) of task blocks, which significantly improved from the first quarter to the fourth quarter. The right panel shows the rates during the fourth quarter under each construct condition (OS, OO, SO, and SS), which are shown in the clockwise order of the two-by-two matrix in Fig. 1. The rates reached 60% (above chance level at 50% denoted by the broken line) for all conditions. Gray bars denote the results under the OS condition as a reference for comparing the four conditions. (**b**) Accuracy rates in the SV task. The rates under the OS condition significantly increased also in the SV task (left). During the fourth quarter, the rates reached 60% except under the SS condition (right). (**c**) The response times (RTs) for each condition during the fourth quarter, shown separately for the GR task (left) and SV task (right). The RTs were comparable among all the conditions in both tasks. (**d**) The d′-values for all participants, indicating a more robust estimation of performances in the GR and SV tasks. We divided these participants into two groups: Groups I and II. (**e**) Accuracy rates in the SV task during each quarter, shown for Group I, who met the criteria of more than 60% (the fourth quarter) in the SV task under both the OS (left) and SO (right) conditions. (**f**) Accuracy rates in the SV task for Group II, who did not reach the above criteria. The rates remained at chance level under the OS (left) and SO (right) conditions. (**g**) The d′-values for Group I (the fourth quarter), which were significantly above chance level at value 0 except for SS in the SV task. (**h**) The d′-values for Group II, none of which were significant. Error bars indicate standard errors of the mean (SEM) for the accuracy rates and RTs, whereas the error bars of d′-values indicate estimated variances. *p < 0.05.

With respect to the GR task, the accuracy rates for all participants steadily increased throughout the block quarters from chance level at 50% to 70% under the OS condition (Fig. 3a, left). Here, we regarded OS as a reference for comparison for the construct conditions (gray bars in Fig. 3). A one-way repeated-measures analysis of variance (rANOVA) indicated a significant main effect of the quarters (*F*(3, 90) = 6.9, *p* = 0.0003), and paired *t*-tests indicated significantly higher rates during the fourth quarter than the first quarter (*t*(30) = 4.8, *p* < 0.0001). With respect to the fourth quarter, the accuracy rates in the GR task reached 60–70% under all the construct conditions (Fig. 3a, right). These rates were significantly above chance level (one-sample *t-*tests, *p* < 0.01, Holm corrected), confirming the above criterial level of 60% for distinguishing Groups I and II. An rANOVA did not indicate a significant difference among the four conditions (*F*(3, 90) = 1.0, *p* = 0.4).

With regards to the SV task, the accuracy rates for all participants also indicated an increase throughout the quarters under the OS condition (Fig. 3b, left). An rANOVA indicated a significant main effect of the quarters (*F*(3, 90) = 6.3, *p* = 0.0006), and paired *t*-tests indicated significantly higher rates during the fourth quarter than the first quarter (*t*(30) = 4.3, *p* = 0.0002). During the fourth quarter, the rates reached 60% except under the SS condition (Fig. 3b, right). An rANOVA indicated a significant main effect of the conditions (*F*(3, 90) = 9.1, *p* < 0.0001), and the rate under the SS condition was significantly lower than that under the OS condition (*t*(30) = 4.8, *p* < 0.0001). Recall that SS was hypothesized to be the most difficult condition (see the *Introduction*). The RTs were comparable for all the construct conditions for each task (rANOVA, *p* > 0.1; Fig. 3c). The accuracy rates in the SV task were most sensitive to reveal performance differences among the construct conditions.

In order to obtain a more robust estimation of performances in both tasks, we employed “the signal detection theory,” which is generally used in order to discriminate the distribution of a signal source that has noise from the distribution of a noise source alone^21^. In doing this, we obtained d′-values as a Z-value of the “hit” rate (i.e., correct detection of ungrammatical and mismatched stimuli in our study) minus that of the “false-alarm” rate (i.e., incorrect responses to grammatical and matched stimuli). In order to examine any significant deviation from chance level (d′ = 0), we estimated variances of d′-values^22^. Although d′-values in the GR task were consistent with the accuracy rates (see Fig. 3a), d′-values in the SV task were significant under the OS and OO conditions alone (*p* < 0.05, Holm corrected for each task). This result was due to the larger variance, i.e., larger individual differences (Fig. 3d). Given that the criterial level noted above (see the *Experimental design* section) was not met by some participants, we divided the participants into Groups I and II according to this criteria during the fourth quarter.

### Group differences in proficiency levels

Consistent with the results for all participants (see Fig. 3b), the accuracy rates for Group I significantly increased from the first to fourth quarters in the SV task under the OS and SO conditions (Fig. 3e), as well as under the OO condition (*p* < 0.05, Holm corrected). In contrast, for Group II, the accuracy rates did not significantly change from the first to fourth quarters under the OS or SO condition (Fig. 3f), nor under the OO or SS condition (*p* > 0.05). The accuracy rates under the SO condition were above 60% during the first quarter. However, this was an exception for Group II, and this tendency immediately dropped to chance level after the second quarter. We confirmed that the actual numbers for the task blocks were comparable for Groups I and II (*t*(29) = 0.6, *p* = 0.5). Considering the notable progress for Group I, we focused on the fourth quarter for subsequent analyses.

For Group I, the d′-values in the GR task were significantly above value 0 under all four conditions, and the d′-values in the SV task were significant under the OS, OO, and SO conditions (*p* < 0.05, Holm corrected for each task; Fig. 3g). In contrast, for Group II, the d′-values under none of the four conditions in the GR or SV task were significantly different from chance level (*p* > 0.05; Fig. 3h). These results confirmed successful building of the sentence structures under the OS, OO, and SO conditions by the Group I participants.

### Event-related group differences in brain activations

**Figure 4:**
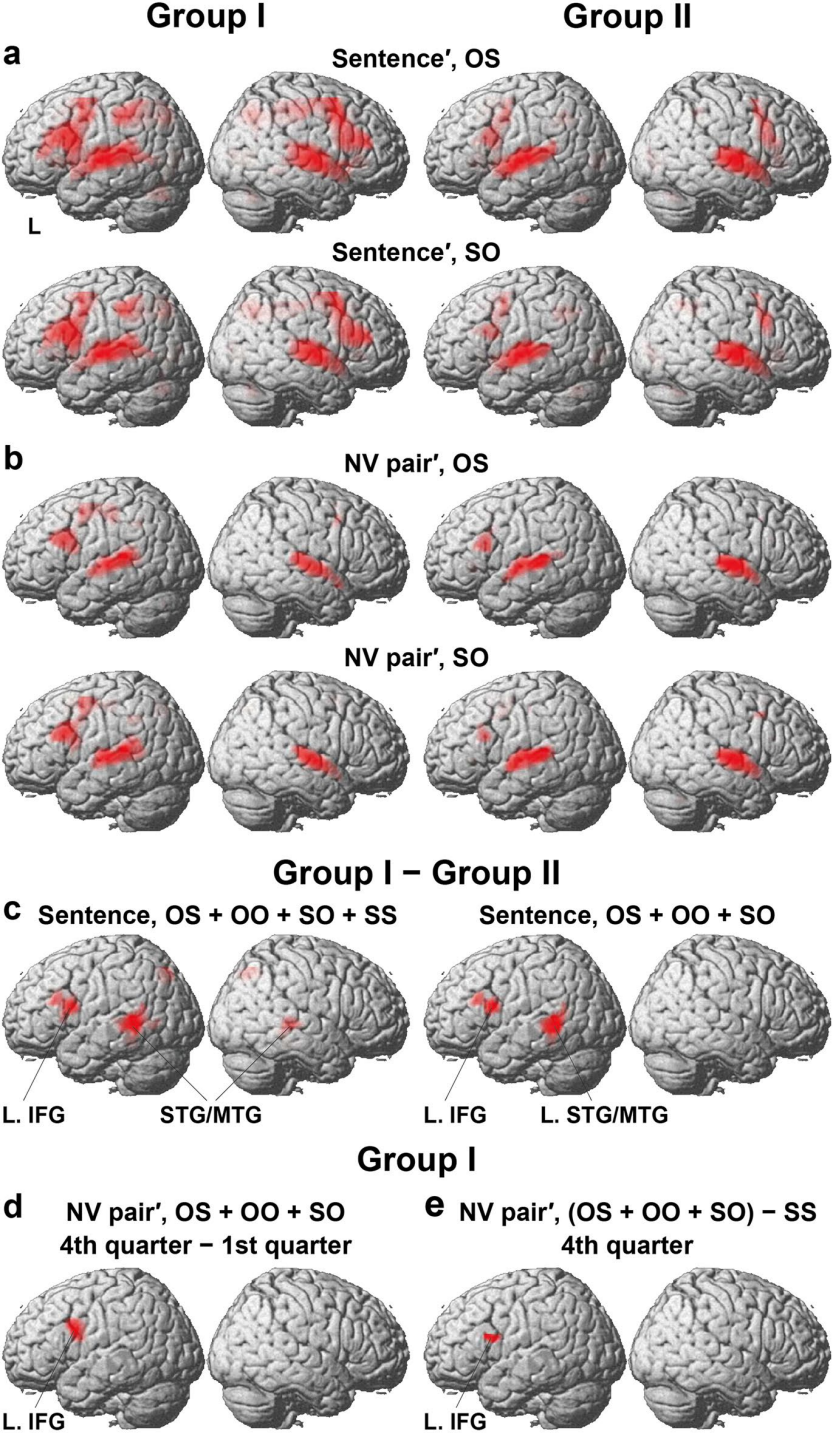
Activations related to groups, phases, and conditions. (**a**) Bilateral activations for each of Groups I and II in the [Sentence − Lexical list] contrast (abbreviated as Sentence′). Activations during the *fourth* quarter are shown under the OS and SO conditions [family-wise error (FWE) corrected *p* < 0.05 for the voxel level]. (**b**) Localized activations for each group in the [NV pair − Lexical list] contrast (abbreviated as NV pair′). The Sentence′ and NV pair′ contrasts were performed in the second-level analyses. (**c**) Focal activations observed in a direct comparison of the [Group I − Group II] contrast (uncorrected *p* < 0.001 for the voxel level and FWE corrected *p* < 0.05 for the cluster level). During the Sentence events, activations were mainly observed in the left inferior frontal gyrus (L. IFG) and superior/middle temporal gyri (STG/MTG) under the OS, OO, SO, and SS conditions (left), or under the OS, OO, and SO conditions (right), consistent with behavioral results (see Fig. 3g). An exclusive mask of negative activation for Group II (one-sample *t*-test, uncorrected *p* < 0.05) was applied. (**d**) L. IFG activations for Group I, observed in the [4th quarter − 1st quarter] contrast. The NV pair′ contrast was performed in the first-level analyses; activations were averaged among OS, OO, and SO conditions. (**e**) L. IFG activations for Group I, further revealed by the [(OS + OO + SO) − SS] contrast during the fourth quarter [uncorrected *p* < 0.001 for the voxel level and false discovery rate (FDR) corrected *p* < 0.05 for the cluster level].

To obtain the overall activation patterns for the entire brain, we compared activations during the “Sentence” event with those during the “Lexical list” event (a list of five words; see Fig. 2b). The Lexical list controlled auditory recognition of individual words used in the stimulus Sentences, as well as for lexico-semantic processing. For all participants, the [Sentence − Lexical list] contrast indicated consistent results under all four construct conditions, revealing *bilateral* activations in the LPMC, dorsal IFG, insula, superior/middle/inferior temporal gyri (STG/MTG/ITG), angular/supramarginal gyri (AG/SMG), and cerebellum VI/Crus I (Supplementary Fig. S3a, for OS and SO). Medial activations were also observed in the supplementary motor area (SMA), anterior cingulate cortex (ACC), basal ganglia, thalamus, precuneus, and calcarine/LG. When Groups I and II were analyzed separately (Fig. 4a), the overall activation patterns for both groups were similar to those for all participants, but the extent of the significant activations was more restricted for Group II in the bilateral LPMC, dorsal IFG, insula, STG/MTG, SMG, and cerebellum, as well as in the medial SMA/ACC, basal ganglia, thalamus, precuneus, and calcarine/LG.

Next, we focused on the NV pair events prior to the SV responses (see Fig. 2b). The extent of activations during the NV pair events was narrowed down in the [NV pair − Lexical list] contrast from those in the [Sentence − Lexical list] contrast for all participants (Supplementary Fig. S3b). When Groups I and II were analyzed separately (Fig. 4b), the overall activation patterns for Group I were similar to those for all participants. We observed activations in the bilateral LPMC, *dorsal left* IFG, bilateral STG/MTG, and L. AG/SMG, as well as in the medial SMA/ACC and thalamus (more than 20 voxels) for Group I (Fig. 4b, for OS and SO; see Table 2 for the list of activated regions under OS). The overall activation patterns were also similar for Group II, but the extent of significant activations was more restricted (see Table 2). These results for both groups confirmed the involvement of language areas and supporting networks during syntactic, semantic, and phonological processes.

**Table 2:** Regions with significant activations related to groups, phases, and conditions.

Brain regions	BA	Side	*x*	*y*	*z*	*Z*	voxel	*x*	*y*	*z*	*Z*	voxel
NV pair − Lexical list, OS			Group I					Group II				
LPMC	6/8	L	− 27	− 1	50	6.0	640	− 45	8	38	4.8	153
			− 30	− 25	59	5.9	*					
IFG	44/45	L	− 51	17	26	6.2	*	− 42	23	20	5.9	*
LPMC	6/8	R	48	8	41	5.4	21	45	11	44	4.6	2
IFG	44/45	R						48	20	26	4.6	2
Insula		L						− 30	29	2	5.0	17
SMA/ACC	6/32	M	− 6	5	53	5.7	114	− 6	11	50	5.1	28
STG/MTG	22/21	L	− 42	− 37	14	7.6	618	− 54	− 10	− 1	7.8	512
			− 51	− 22	5	7.3	*	− 54	− 49	14	5.2	*
			− 63	− 37	11	5.7	*					
STG/MTG	22/21	R	54	− 13	− 1	7.7	413	54	− 13	2	> 8	429
Temporal pole	38	R	54	11	− 13	5.6	*					
AG/SMG	39/40	L	− 36	− 49	41	5.4	29					
Precuneus	7	M	− 12	− 73	50	4.7	3					
Thalamus		M	− 6	− 22	− 7	5.5	22	6	− 22	− 13	4.7	6
Cerebellum VI/Crus I		L	− 30	− 70	− 31	5.1	4					
Cerebellum VI/Crus I		R	27	− 64	− 31	4.6	2					

Group I − Group II, Sentence			OS + OO + SO + SS					OS + OO + SO				
IFG	44/45	L	− 51	26	26	5.6	194	− 51	26	26	5.1	152
	44/45/6	L	− 57	11	17	5.5	*	− 57	11	17	5.0	*
STG/MTG	22/21	L	− 57	− 43	− 1	> 8	333	− 57	− 43	− 1	7.3	241
			− 57	− 64	2	3.9	*					
MTG/ITG	21/20	L	− 48	− 40	− 16	4.5	*	− 48	− 40	− 16	4.2	*
STG/MTG	22/21	R	54	− 31	2	5.6	165					
MTG/ITG	21/20	R	42	− 34	− 16	4.0	*					
Precuneus	7	M	− 12	− 76	50	5.8	308					
			9	− 70	53	4.6	*					

Group I, NV pair − Lexical list, OS + OO + SO, 4th quarter − 1st quarter												
IFG	6/44/45	L	− 51	5	29	4.5	161					

Group I, NV pair − Lexical list, (OS + OO + SO) − SS, 4th quarter												
IFG	44/45/6	L	− 51	14	20	3.8	41					

Stereotactic coordinates (*x*, *y*, *z*) in the MNI space are shown for activation peaks of *Z* values, which were more than 16 mm apart (see Fig. 4). The region with an asterisk is included within the same cluster with the region shown in the row right above. BA: Brodmann’s area; L: left; M: medial; R: right; ACC: anterior cingulate cortex; AG: angular gyrus; LPMC: lateral premotor cortex; IFG: inferior frontal gyrus; SMA: supplementary motor area; SMG: supramarginal gyrus; STG/MTG/ITG: superior/middle/inferior temporal gyri.

Following qualitative comparisons between the two groups, we performed a *direct* group comparison, i.e., directly obtaining the functional map of the [Group I − Group II] contrast. We focused on the Sentence events, averaged among the four conditions. Significant activations were observed in the dorsal L. IFG and bilateral STG/MTG (Fig. 4c, left), as well as in the medial precuneus. These regions were subsections of the activated regions for Group I in the [Sentence − Lexical list] contrast (see Fig. 4a).

Given that the participants in Group I performed better in the OS, OO, and SO conditions than in the SS condition (see Fig. 3g), we next focused on the former three conditions. We repeated the same group comparison and observed more localized activations in the dorsal L. IFG and L. STG/MTG alone (Fig. 4c, right; Table 2).

### Condition-specific temporal activation changes

Following the direct group comparisons shown above, we next focused on Group I. To determine which of the above-mentioned regions were critical for the final phase in our testing, we directly compared the activations between the initial and final phases, i.e., the [4th quarter − 1st quarter] contrast. We focused on the OS, OO, and SO conditions in the [NV pair − Lexical list] contrast. We observed focal activation in the dorsal L. IFG alone (Fig. 4d, Table 2), indicating temporal activation changes occurring continuously from the initial to final phases.

We further examined the activations specific to the successful construct conditions. For the contrast [(OS + OO + SO) − SS], focal activation was observed also in the dorsal L. IFG (Fig. 4e, Table 2). This region mainly consisted of Brodmann’s areas (BA) 44/45, and included more BA 45 than the region shown in Fig. 4d. With regards to the three regions of the dorsal L. IFG, observed in the separate analyses (see Fig. 4c–e), we confirmed an overlap of eight significant voxels among these clusters. For Group II, neither of the [4th quarter − 1st quarter] or [(OS + OO + SO) − SS] contrasts showed any significant activation. These results further confirmed the central role of the dorsal L. IFG in successful structure building processes.

We also conducted two-way [groups × quarters] analyses of covariance (rANCOVAs) under the OS, OO, and SO conditions. We observed focal activations in the dorsal L. IFG for the main effect of quarters in the [NV pair − Lexical list] contrast (Supplementary Fig. S3c), replicating the results of Group I (Fig. 4d) for all participants. Moreover, we also found a significant interaction of groups by quarters in the [Sentence − Lexical list] contrast (Supplementary Fig. S3d).

### Brain activations related to the subsequent task performance

**Figure 5:**
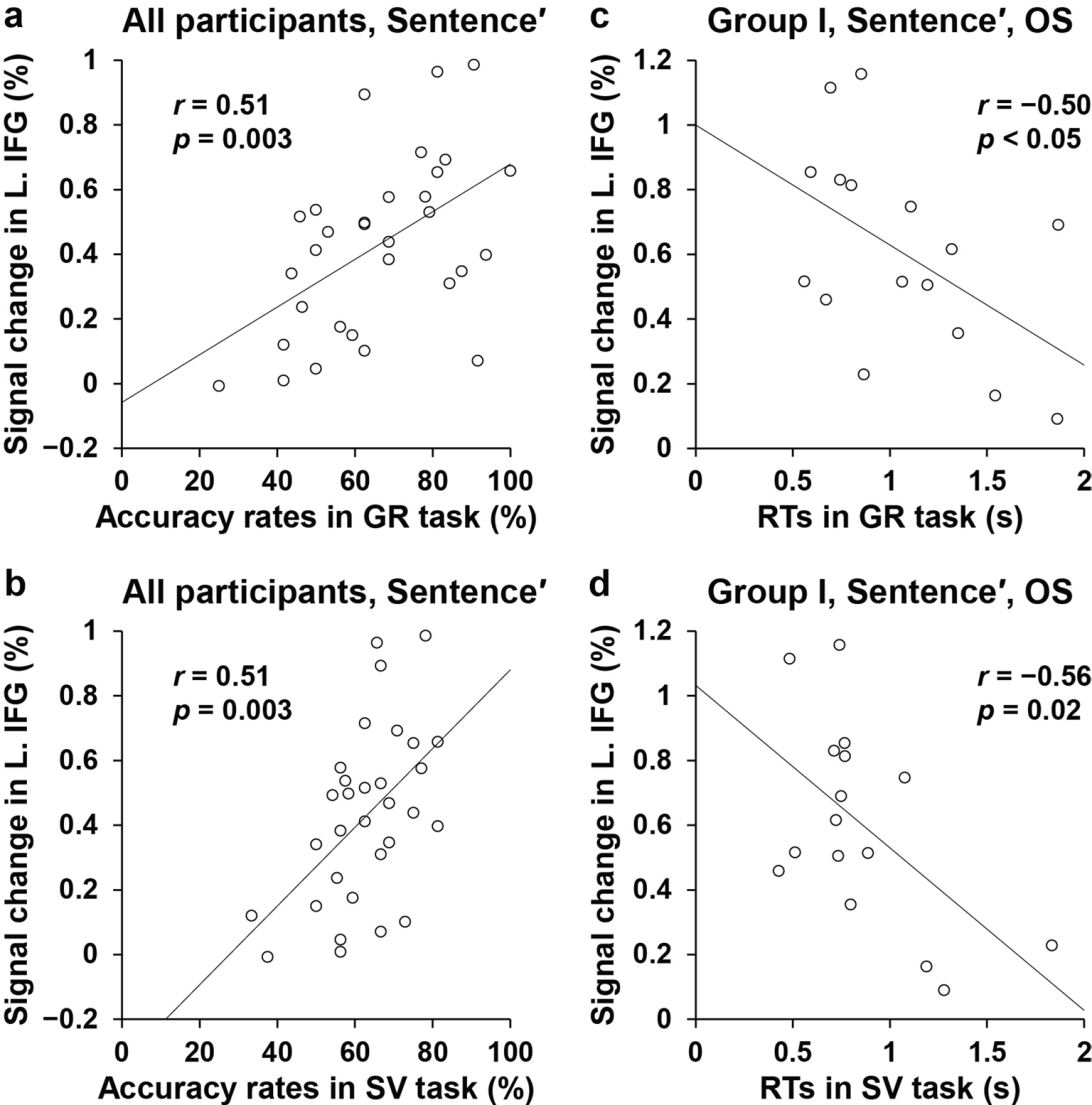
Brain activations related to the subsequent task performances. (**a**) A correlation between L. IFG activations and accuracy rates in the GR task for all participants. Averaged among the four construct conditions, the rates in the GR task became higher for the participants, who showed stronger activations in the Sentence′ contrast. The region of interest (ROI) for this figure was determined by the activated region in Fig. 4e. (**b**) A similar correlation in the SV task. Stronger L. IFG activations also predicted higher rates in the SV task. (**c**) A correlation between L. IFG activations and RTs in the GR task for Group I. Under the OS condition, the RTs became shorter indicating higher task proficiencies, for the participants with stronger activations. (**d**) A similar correlation in the SV task. Stronger L. IFG activations again predicted shorter RTs. Error bars indicate SEM.

For all the participants, we conducted additional region of interest (ROI) analyses for the cluster of the dorsal L. IFG activations, identified by the [(OS + OO + SO) − SS] contrast in Fig. 4e. We focused on the signal changes in the [Sentence − Lexical list] contrast, in order to examine whether those reliably enhanced activations affected the *subsequent processes* required by a grammatical judgment about the Sentence, and by the correct identification of the SV pairs. Averaged among the four conditions, significantly positive correlations were observed between accuracy rates and dorsal L. IFG activations for the GR (Fig. 5a) and SV (Fig. 5b) tasks (both, *r* = 0.51, *p* = 0.003).

We also focused on the OS condition for Group I, and observed significantly *negative* correlations between RTs and dorsal L. IFG activations for the GR (Fig. 5c) and SV (Fig. 5d) tasks (GR task: *r* =  − 0.50, *p* < 0.05; SV task: *r* =  − 0.56, *p* = 0.02). These results demonstrate that higher signal changes in the dorsal L. IFG measured during the Sentence events actually *predicted* higher accuracy rates and shorter RTs for the subsequent experimental tasks.

## Discussion

In our previous study^6^, we examined the initial acquisition of Kazakh sentences under three step-wise grammar conditions with distinct sentence structures: a *conjoined* sentence, a *nested* sentence, and a sentence involving a *relative* clause. As a next step in the present study, participants were presented with sentences that consisted of main and relative clauses that both included nouns as *objects*. The inclusion of *objects* was newly introduced in this phase of the design, which made the tested conditions very demanding. Moreover, the four types of sentence structures (OS, OO, SO, and SS; Fig. 1) were presented to the participants in a completely randomized order. By simply alternating demo and task trials (Fig. 2), we were able to test participants on their abilities to build sentence structures in a new language without the explicit teaching of grammatical rules.

For the analyses with respect to the behavioral and functional data, we divided the participants into Groups I and II based on the levels attained on the subject-verb matching task. We obtained the following three results. First, consistent with successful building of complex sentence structures under the OS, OO, and SO conditions for Group I (Fig. 3g), but not for Group II (Fig. 3h), the contrast of [Group I − Group II], which examined those participants *who* parsed the structures, indicated that the dorsal L. IFG and L. STG/MTG, the core language areas, were significantly activated under the OS, OO, and SO conditions (Fig. 4c). Secondly, focusing on Group I, the contrast of [4th quarter − 1st quarter], which examined *when* the structures were parsed (Fig. 4d), as well as that of [(OS + OO + SO) − SS], which examined *what* structures were parsed (Fig. 4e), additionally demonstrated focal activations in the dorsal L. IFG alone. Thirdly, among the individual participants, stronger activation in the dorsal L. IFG, measured during the Sentence events, predicted higher accuracy rates and shorter RTs for the execution of each of the tasks that followed (Fig. 5). These results cannot be explained by task difficulty or memory loads, and they, instead, indicate a critical and consistent role of the dorsal L. IFG during the initial to intermediate stages of grammar acquisition in a new target language. Such functional specificity of the dorsal L. IFG, i.e., the grammar center, provides neuroscientific evidence consistent with the claims made by the CEM in investigating language acquisition beyond target L2/L3s.

The results observed with respect to “*what* structures were parsed” support the claim that the dorsal L. IFG played an essential role in syntactic processing as used for successful building of sentence structures in a new target language. While left ventral BA 44 has been associated with syntactic structure building^23^, left dorsal BA 44 and the inferior frontal sulcus have been suggested to be linked to memory loads^24,25^. In the present study, however, we demonstrated that activations in the dorsal L. IFG were free from task difficulty or memory loads, because larger activations were observed in the participants indicating less difficulty in both GR and SV tasks (Fig. 5). Moreover, when the dorsal L. IFG, together with the L. LPMC, was damaged by a glioma, we have already reported clear evidence of agrammatic comprehension^19,26^. In those studies, we used a picture-sentence matching task that involved no memory load. It should be also noted that there were wide individual variations in the extent of BA 44, as reported in a previous anatomical study, which stated that “the volumes of area 44 differed across subjects by up to a factor of 10”^27^. Therefore, we did not separate the dorsal L. IFG into BAs 44, 45 and 6. The eight voxels overlapped among the three clusters (Fig. 4c–e) were located in BAs 44/6.

Regarding the “*who*,” the overall activations were weaker and spatially more restricted for Group II than for Group I. In Group II, the proficiency improvement from the initial phase was absent during the experimental testing, while it was present in Group I (Fig. 3e–h). Moreover, brain activations reflected individual differences in proficiency levels in terms of the two groups (Fig. 4a–c). This might represent a prior developmental phase for those participants in Group II or might be a developmental “delay” in comparison to Group I, although the explanation for these hypotheses is beyond the scope of this paper. Another possibility is that the significant group differences may originate from difficulty in the processing of the phonology/phonetics and formal semantics simultaneously. This possibility is supported by the bilateral STG/MTG activations in the direct group comparison (Fig. 4c), which were stronger in the L. STG/MTG (included in Wernicke’s area), the core region for phonological processes^10^. However, activations in the dorsal L. IFG (included in Broca’s area), the core region for syntactic processes, also indicate that the crucial factor was constructing phrase-level structures, substantiating “the basic property of language”^28^. The constraints and mechanisms involved in language acquisition, accompanied by substantial individual differences, require further elucidation.

We observed bilateral activations in the LPMC during the Sentence events, which were stronger for Group I than for Group II (Fig. 4a). Moreover, more localized activations in the L. LPMC were evident during the NV pair events, also stronger for Group I (Fig. 4b). Our previous fMRI studies on grammatical judgments have consistently reported activations in the dorsal L. IFG and L. LPMC^6,10,19,29–31^. Our recent fMRI study explicitly tested subject-predicate correspondence for sentences in L1, and revealed critical activations in the bilateral LPMCs for processing *dependencies* solely determined by hierarchical structures, when compared with those based on linear sequences of words^32^. These findings provide additional support for the results of other neuroimaging experiments^7,33–35^.

A number of case studies on aphasic patients have commonly and simplistically identified the crucial roles of Wernicke’s area as pertaining to input/comprehension alone, and those of Broca’s area as output/production alone^36^. However, if we assume that these regions are responsible for not only loss but also acquisition of a language specific grammar, in line with the present study, it is necessary to revise the classical notions of Wernicke’s and Broca’s areas with respect to language processing^37^. Moreover, neuroimaging studies have identified the dorsal L. IFG as a core hub for the computation of linguistic information for both signed and verbal languages—each of which uses a different modality for externalization^38–40^. Given the clear distinction between the core language system and external sensory-motor systems^28^, we conclude that the core system of the dorsal L. IFG is independent from both sensory input and motor output.

In line with proposals that the same principles constrain L1 and L2 acquisition^5^, as well as for L3^41^ and subsequent L4, …, L*n*, we predicted that the dorsal L. IFG, the grammar center, becomes functional during acquisition of a language-specific grammar for any new language, suggesting an essential and universal property about human linguistic capacity, which enables both unlimited acquisition and use of multiple languages. To conclude, the current neuroscientific evidence for a grammar acquisition of an L4, together with the CEM which is hypothesized to *account for* (i.e., the “*why*”) the mechanisms that universally underlie language acquisition, provides further essential insight critical for clarification concerning what is involved in successful building of sentence structures in a new language.

## Materials and methods

For more details, see the Supplementary Methods.

### Participants

Volunteers, who were native speakers of Japanese, were recruited from multiple sources for this study; these included the LEX Institute (Hippo Family Club), the University of Tokyo, and Sophia University. Thirty-three participants, in total, met the criteria set for the G1-G3 conditions (as described in the* Introduction*) and reached G4. Right-handedness was estimated as a laterality quotient (LQ) according to the Edinburgh inventory^42^. Because of their left-handedness (i.e., negative LQ), two participants were eliminated from the analyses. We divided the resultant 31 participants into two groups (see the *Introduction* for the criteria): Group I [16 participants; nine multilinguals and seven bilinguals] and Group II [15 participants; eight multilinguals and seven bilinguals] (see Supplementary Table S2). There was no group difference in duration of exposure (DOE) to English, Avant score (i.e., language proficiency level) in English, and LQ (*p* > 0.2). The mean age was significantly lower for Group II (*t*(29) = 2.3, *p* = 0.03). Note that Groups I and II included one and five participants under the age of 19, respectively; for those participants above age 19, age was not significantly different (*t*(23) = 1.7, *p* = 0.1). Age was thus used as a nuisance factor in the activation analyses (see Supplementary Methods, *fMRI data analyses*). None of the participants in the study had neurological or psychiatric disorders.

Prior to their participation in the study, the nature and possible consequences of the study were explained to each participant and written informed consent was obtained immediately after this introduction. Approval for the experiments was obtained from the ethical review board of experimental studies on human subjects at Graduate School of Arts and Sciences, the University of Tokyo (No. 464). All research was performed in accordance with the Declaration of Helsinki, Singapore Statement on Research Integrity, and relevant guidelines/regulations in Japan (Science Council of Japan, and Japan Society for the Promotion of Science). This clinical trial has been registered in a publicly accessible primary register at Japan Registry of Clinical Trials (jRCT) on 25/12/2020 (No. jRCT1030200294).

### Stimuli

Auditory stimuli in Kazakh consisted of 76 sentences (44 grammatical and 32 ungrammatical) with a limited number of lexical items shown in Supplementary Table S3. The stimulus sentences were recorded by a male native speaker of Kazakh, and individual words were also separately recorded. Both grammatical and ungrammatical sentences were articulated at a somewhat slower pace for the participants, who were not familiar with Kazakh. By using the Wavelab 8 software (Steinberg Media Technologies GmbH, Hamburg, Germany), we digitized the stimuli (16 bit, 44.1 kHz, stereo), where the maximum volume of each stimulus was equally set to − 1 dBFS. The duration of all the stimulus sentences was adjusted to 4.75 s (see Fig. 2b), maintaining the original pitch of the sentences. As a lexical reference, five words (“Lexical list”) used in the sentence were presented auditorily at the beginning of each task trial; individual words translated into English were visually presented (see Fig. 2b). The English translations provided a clue to meanings and parts of speech for the Kazakh words. During the MR scans, the participants wore a set of MRI-compatible headphones (Resonance Technology Inc., Northridge, CA), a pair of earmuffs (3 M Peltor, St. Paul, MN), and a pair of earplugs (Earasers, Persona Medical, Casselberry, FL) to reduce the high-frequency noises (> 1 kHz) of the scanner.

### Tasks

Each of demo and task blocks consisted of two trials each for four construct conditions (OS, OO, SO, and SS) in a randomized order. We used four trials per demo block for the G1-G3 conditions but increased the number of trials to eight for the most demanding G4 condition. Different sets of sentences were used for the demo and task blocks to avoid simple memorization of the stimuli sentences by the participants. Although the participants were inside the scanner, we conducted a demo block when there was no scanning being conducted; during this exposure period, the auditory stimuli were presented without the loud noise involved in MR scanning. In a demo block, the first two sentences were always grammatical, and the remaining six sentences consisted of four grammatical and two ungrammatical sentences presented in a randomized order. In a task block when there was scanning, both grammatical and ungrammatical sentences were completely randomized. Regarding the NV pairs in the demo block, the first two pairs were always matched; a mismatched pair never followed an ungrammatical sentence. Otherwise, matched, and mismatched pairs were randomized. In a task block, both matched and mismatched pairs were completely randomized.

During the experiments, we used the same criteria as those used for G1-G3 for the G4 condition, such that each participant correctly performed at least six out of the eight task trials in each of two blocks (not necessarily consecutive) for both the GR and SV tasks. With one or two days of experimentation, 15 out of the 31 participants reached these criteria. Among the remaining 16, five out of ten participants who correctly performed at least six out of the eight task trials in one block for both the GR and SV tasks were further tested on another day. Three out of those five participants reached the criteria on the third day. In the end, there were between 5 and 28 task blocks (i.e., 40–224 task trials) depending on the participant, and thus between 2 and 7 task blocks for the fourth quarter. After each block of task trials, the participants were informed of the number of their correct responses (e.g., 6 out of 8) separately for the GR and SV tasks. In each scanning run, we added five task trials under the Words condition (see Supplementary Methods, *Tasks*).

### MRI data acquisition and analyses

The MRI scans were conducted in a 3.0 T scanner (Signa HDxt; GE Healthcare, Milwaukee, WI) with a bird-cage head coil. Each participant was in a supine position, and his or her head was immobilized inside the coil. With respect to the structural images, high-resolution T1-weighted images of the whole brain [136 axial slices, 1 × 1 × 1 mm^3^] were acquired with a three-dimensional fast spoiled gradient-echo (3D FSPGR) acquisition [repetition time (TR) = 8.6 ms, echo time (TE) = 2.6 ms, flip angle (FA) = 25°, field of view (FOV) = 256 × 256 mm^2^]. With respect to the fMRI time-series data we used a gradient-echo echo-planar imaging (EPI) sequence [TR = 2 s, TE = 30 ms, FA = 78°, FOV = 192 × 192 mm^2^, resolution = 3 × 3 mm^2^]. We scanned a set of 30 axial slices that were 3-mm thick with a 0.5-mm gap, covering the range of −38.5 to 66 mm from the line of the anterior commissure to posterior commissure (AC-PC). In a single scanning session, we obtained 145 volumes, and dropped the initial four volumes from analyses due to MR signal increases. The fMRI data were analyzed in a standard manner using SPM12 statistical parametric mapping software (Wellcome Trust Center for Neuroimaging, http://www.fil.ion.ucl.ac.uk/spm)^43^ implemented on MATLAB (Math Works, Natick, MA). For the fMRI data analyses, we used all trials including both correctly and incorrectly answered trials in order that we would be able to examine the activations that reflected accuracy rates directly for the tasks (see Fig. 5a, b); all conditions tested were equally weighted regarding the number of trials administered. See the Supplementary Methods for details.

### Supplementary Information

Supplementary Information.

## Data Availability

The datasets generated during and/or analyzed during the current study are available from the corresponding author upon reasonable request.
