# Supplementary material for: Enhanced activations in the dorsal inferior frontal gyrus specifying the who, when, and what for successful building of sentence structures in a new language
Source: Sci Rep. 2024 Jan 2;14:54. doi: 10.1038/s41598-023-50896-6 (PMC10761922; doi:10.1038/s41598-023-50896-6)
Supplement: Supplementary file 1 — Supplementary Information. [file 41598_2023_50896_MOESM1_ESM.pdf]

# Enhanced activations in the dorsal inferior frontal gyrus specifying the *who*, *when*, and *what* for successful building of sentence structures in a new language

**Keita Umejima[1], Suzanne Flynn[2], and Kuniyoshi L. Sakai[1,*]**

[1] Department of Basic Science, Graduate School of Arts and Sciences, The University of Tokyo, 3-8-1 Komaba, Meguro-ku, Tokyo 153-8902, Japan
[2] Department of Linguistics and Philosophy, Massachusetts Institute of Technology, 77 Massachusetts Avenue, 32-D808, Cambridge, MA 02139, USA

*Correspondence: Department of Basic Science, Graduate School of Arts and Sciences, The University of Tokyo, Komaba, 3-8-1 Komaba, Meguro-ku, Tokyo 153-8902, Japan. Fax: +81-3-5454-6261. *E-mail*: sakai@sakai-lab.jp (K.L. Sakai).

**Supplementary Table S1. Language background of the participants.**

| No. | Age | L2: English | | | L3 | | | | L4 | | | L5 | | |
|---|---|---|---|---|---|---|---|---|---|---|---|---|---|---|
| | | DOE (yr.) | DOR (mo.) | Avant Score | Lg. | DOE (yr.) | DOR (mo.) | Avant Score | Lg. | DOE (yr.) | DOR (mo.) | Lg. | DOE (yr.) | DOR (mo.) |
| 1 | 20.5 | 10.1 | | 2 | ZH | 2.1 | | | | | | | | |
| 2 | 16.5 | 5.1 | | 4 | FR | 1.2 | | | | | | | | |
| 3 | 18.3 | 4.2 | 13 | 7 | | | | | | | | | | |
| 4 | 19.2 | 8.3 | | 4 | KO | 0.2 | | | | | | | | |
| 5 | 24.6 | 12.3 | | 5 | DE | 6.3 | | | | | | | | |
| 6 | 14.9 | 4.3 | | 4 | | | | | | | | | | |
| 7 | 26.3 | 13.8 | | 5 | ZH | 7.9 | | | | | | | | |
| 8 | 23.6 | 11.0 | | 4 | DE | 4.9 | | | | | | | | |
| 9 | 21.5 | 11.0 | | 4 | DE | 1.9 | | | RU | 1.0 | | | | |
| 10 | 21.8 | 9.0 | | 4 | IT | 3.0 | | | | | | | | |
| 11 | 22.5 | 9.9 | | 9 | ES | 3.0 | | | | | | | | |
| 12 | 23.8 | 11.0 | | 4 | ZH | 3.0 | | 1 | | | | | | |
| 13 | 21.3 | 9.0 | | 4 | ES | 3.0 | | | | | | | | |
| 14 | 20.7 | 16.0 | | 6 | ZH | 2.0 | | | | | | | | |
| 15 | 24.1 | 11.1 | 5 | 5 | ES | 3.1 | 9 | 5 | PT | 5.7 | 10 | | | |
| 16 | 25.3 | 19.4 | 4 yr. | 9 | ES | 9.3 | 11 | 8 | | | | | | |
| 17 | 18.5 | 12.5 | | 5 | ES | 2.2 | 11 | 5 | | | | | | |
| 18 | 25.0 | 18.6 | | 7 | ES | 9.3 | 10 | 5 | ZH | 5.6 | 4 | | | |
| 19 | 20.2 | 11.7 | | 4 | ES | 2.8 | 10 | 3 | KO | 0.7 | | | | |
| 20 | 22.0 | 13.6 | | 4 | ES | 4.3 | 10 | 4 | | | | | | |
| 21 | 20.5 | 10.6 | | 4 | ES | 3.3 | 11 | 5 | KO | 0.6 | | | | |
| 22 | 23.3 | 10.6 | | 4 | ES | 6.3 | 11 | 3 | | | | | | |
| 23 | 20.4 | 7.6 | | 8 | ES | 2.1 | 9 | 5 | | | | | | |
| 24 | 22.4 | 13.9 | 11 | 6 | ES | 0.8 | 9 | 5 | FR | 3.9 | | | | |
| 25 | 19.5 | 7.0 | | 4 | ES | 1.5 | 10 | 3 | | | | | | |
| 26 | 18.3 | 6.1 | | 4 | ES | 1.3 | 10 | 2 | | | | | | |
| 27 | 19.6 | 7.7 | | 6 | ES | 0.7 | | 3 | | | | | | |
| 28 | 16.2 | 9.2 | 2 | 4 | ES | 0.2 | | 2 | | | | | | |
| 29 | 19.8 | 7.1 | | 4 | RU | 2.1 | | 6 | | | | | | |
| 30 | 23.6 | 11.2 | | 4 | DE | 5.3 | | 3 | ES | 2.3 | | ZH | 0.9 | |
| 31 | 21.6 | 9.9 | | 7 | DE | 2.9 | | 3 | IT | 2.8 | | ZH | 2.6 | |

The participants shown in Figs. 3a-d are listed here as a subset of the participants reported on in our previous study [1]. Participants 1-14 were bilinguals and Participants 15-31 were multilinguals in that study; see our previous paper [1] for details including the definition of bilinguals/multilinguals. In the present study, there were no statistical differences between the bilinguals and multilinguals under the G4 condition regarding the behavioral results or brain activations. The table above shows the number of languages (second to fifth languages, L2-L5) that each participant had exposure to, if any were known. Language proficiency in English was evaluated by the *Listening Comprehension* sub-test of the Avant STAMP 4S (Standards-based Measurement of Proficiency - 4 Skills). This English proficiency test was administered to all participants. With respect to the other languages, participants first received a sample test for at least one language other than English, and those who were able to answer the first question in this sample test then received the full test. If a participant stopped taking the sample test, the score was not registered. DOR: duration of residence in a country where the language is an official language; DOE: duration of exposure to each language; Lg.: language; DE: German; ES: Spanish; FR: French; IT: Italian; KO: Korean; PT: Portuguese; RU: Russian; ZH: Chinese.

**Supplementary Table S2. Basic data for participants.**

| Group | N | Age (yr.) | DOE (yr.) | Avant score | LQ |
|---|---|---|---|---|---|
| I | 16<br>(7 females) | 22.2 ± 2.6<br>(14.9-26.3) | 10.8 ± 3.3<br>(4.3-18.6) | 5.3 ± 1.7<br>(4-9) | 93 ± 11<br>(71-100) |
| II | 15<br>(7 females) | 20.1 ± 2.6<br>(16.2-25.3) | 10.0 ± 4.0<br>(4.2-19.4) | 4.7 ± 1.7<br>(2-9) | 87 ± 18<br>(47-100) |

For each group, averaged data (mean ± standard deviation) and the ranges for these data (in parentheses) are shown. The Avant score indicates the English proficiency level as assessed by the Listening Comprehension sub-test from the Avant tests (scores 1-9). N: number of participants; DOE: duration of exposure to English; LQ: laterality quotient.

**Supplementary Table S3. Lexical items from Kazakh used in this study.**

| Verbs | | | Translation in English |
|---|---|---|---|
| | Adjectival participle | | |
| Simple past | Perfect | Habitual past | |
| *tüsindi* | *tüsingen* | | *Understood* |
| *oyladï* | *oylaɣan* | | *Thought* |
| *tanïdï* | | *tanïytïn* | *Recognized* |
| *bildi* | | *biletin* | *Knew* |

| Nouns | | |
|---|---|---|
| Nom. | Acc. | |
| *biz* (first pl.) | *Bizdi* | *we*/*us* |
| *siz* (second sg.) | *Sizdi* | *you*/*you* |
| *ol* (third sg.) | *Onï* | *he*/*him* |
| *John* | *Johndï* | *John*/*John* |
| *Dan* | *Dandï* | *Dan*/*Dan* |
| *adam* | *Adamdï* | *man*/*man* |

In our experiments, we used four verbs and six nouns (three pronouns, two proper nouns, and one common noun). nom.: nominative; acc.: accusative; sg.: singular; pl.: plural.

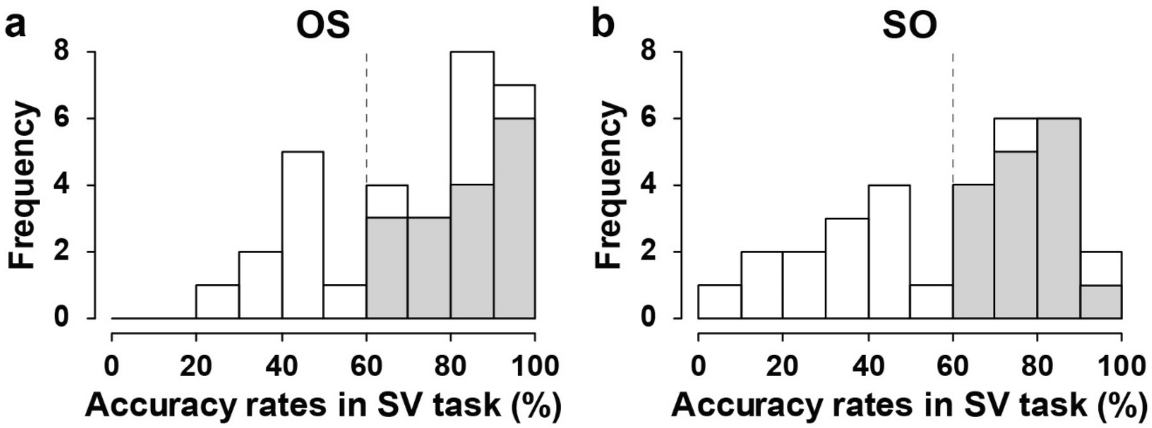


**Supplementary Figure S1. A bimodal distribution for accuracy rates under each of the OS (a) and SO (b) conditions.** The number of participants were plotted against accuracy rates in the SV task during the 4th quarter. The broken lines at 60% indicate the transition point between the two peaks. The gray portions indicate the participants from Group I.

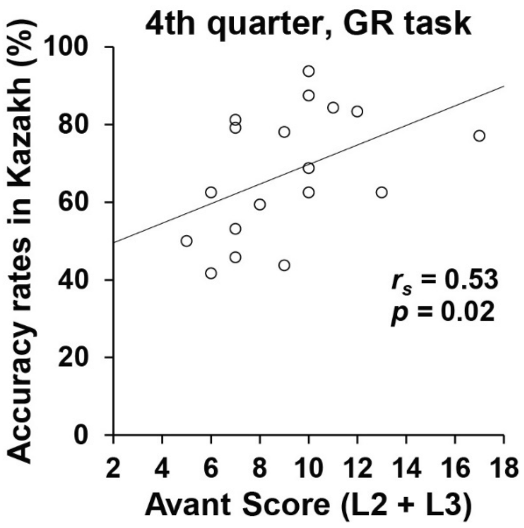


**Supplementary Figure S2. A correlation between performances in Kazakh and the L2/L3.** This correlation was obtained for all participants who received the Avant tests in both the L2 and L3 (see Supplementary Table S1). Higher Avant scores, i.e., higher language proficiency levels in the L2 and L3, predicted higher accuracy rates in the GR task during the fourth quarter. The rates were averaged among the four construct conditions.

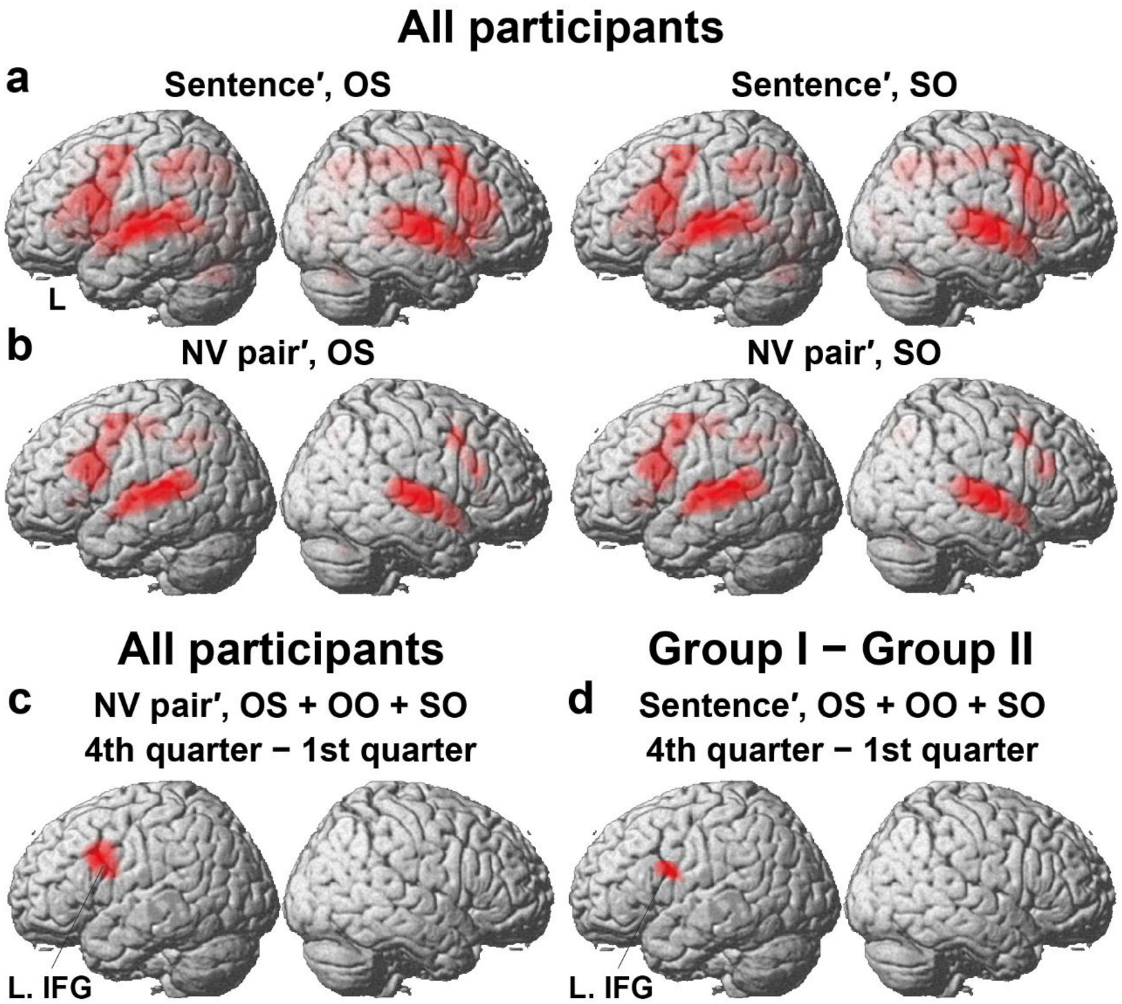


**Supplementary Figure S3. Global and local syntax-related activations.** **(a)** Bilateral activations for *all participants* in the [Sentence − Lexical list] contrast (abbreviated as Sentence′). Activations during the fourth quarter are shown under the OS and SO conditions [family-wise error (FWE) corrected $p < 0.05$ for the voxel level]. **(b)** Localized activations in the [NV pair − Lexical list] contrast (abbreviated as NV pair′). The Sentence′ and NV pair′ contrasts were performed in the second-level analyses. **(c)** L. IFG activations for *all participants* revealed by the main effect of quarters in a two-way [groups × quarters] rANCOVA (uncorrected $p < 0.001$ for the voxel level and FWE corrected $p < 0.05$ for the cluster level). The NV pair′ contrast was performed in the first-level analyses; activations were averaged among OS, OO, and SO conditions to replicate the results of Group I (Fig. 4d). An exclusive mask of negative activation for the 1st quarter was applied (one-sample *t*-test, uncorrected $p < 0.05$). **(d)** L. IFG activations revealed by the interaction of a two-way [groups × quarters] rANCOVA (uncorrected $p < 0.001$ for the voxel level and FWE corrected $p \leq 0.05$ for the cluster level). The Sentence′ contrast was performed in the first-level analyses; activations were averaged among OS, OO, and SO conditions.

**Supplementary Methods**

***The Kazakh vocabulary used in this study***

Ten words (Supplementary Table S3) were chosen for the experimental design; they consisted of four verbs (*tüsindi* "*understood*," *oyladï* "*thought*," *tanïdï* "*recognized*," and *bildi* "*knew*"), three pronouns (*biz* "*we*," *siz* "*you*," and *ol* "*he*"), two proper nouns (*John* and *Dan*), and one common noun (*adam* "*man*"). With respect to the verbs, we used the simple past tense form (e.g., *tüsindi* and *tanïdï*; see Supplementary Table S3, verbs), as well as the perfect form (e.g., *tüsingen*) and habitual past form (e.g., *tanïytïn*) resulting in an adjectival participle. With respect to the nouns, their suffixes change according to their syntactic case in the stimulus sentence. An accusative form (e.g., *adamdï* and *Johndï*; see Supplementary Table S3, nouns) is constructed by adding the suffix allomorph *-di*/*-dï* to its nominative form, except for *ol* which utilizes an accusative form *onï*. With respect to the selection between *-di*/*-dï*, Kazakh suffix allomorphs are determined in accordance with the rules of vowel harmony for each word.

In this experiment, we used the following syntactic subjects: the first-person plural *biz*, second-person singular (polite form) *siz*, and third-person singular *ol*, *John*, *Dan*, and *adam*. In addition, the verbs in the simple past tense utilize personal suffixes according to the person and number of the subject (i.e., SV agreement). According to the rules of vowel harmony in Kazakh, the personal suffix of a verb becomes the allomorphs *-dik*/*-dïq* for the subject *biz* (first-person plural), and *-diŋiz*/*-dïŋïz* for the subject *siz* (second-person singular). For the subject of a third-person singular (*ol*, *John*, *Dan*, and *adam*), the personal suffix of a verb becomes the allomorphs *-di*/*-dï*. We used stimulus sentences, in which the main verb was in the simple past tense, whereas the subordinate verb was an adjectival participle. In Kazakh, the adjectival participle has a fixed suffix regardless of the person and number of the subject

noun phrase. The stimulus sentences were checked for grammaticality and accuracy by native speakers of Kazakh.

***Stimuli***

Under the G4 condition, each trial began with a visual cue of "G4" (Fig. 2). In a *task* trial, this cue was followed by the "Lexical list" of five Kazakh words: two verbs (in the simple past tense with a third-person singular suffix), two nouns (a pronoun and a proper noun; in the nominative case), and *adamdï* (or *adam*). This order of presentation was maintained throughout all trials. Given that the verbs used in the study had generally a greater number of syllables than did the nouns, we adjusted the stimulus length of a verb and that of a noun to 0.6 s and 0.5 s, respectively; the original pitch for each word was maintained throughout. For the Lexical list, each of the five Kazakh words presented *auditorily* was accompanied by a *visual* presentation of an English translation of the Kazakh word (see Fig. 2b). Each visual presentation started 0.25 s before the auditory presentation, and continued until 0.25 s after the end of the auditory presentation. We further added an interval of 0.5 s between each visual stimulus.

In each demo trial (Fig. 2a), a five-word sentence ("Sentence" capitalized here) was presented auditorily. A visual sign (either + or −) was simultaneously presented for 5.0 s, indicating the grammaticality of the sentence (see Table 1), either grammatical (+) or ungrammatical (−); an ungrammatical sentence always included an error in the verb suffix. We then randomly extracted an "NV pair" from the Sentence in the same trial. After a pause of 2.7 s, the NV pair was presented auditorily with a silent period of 1 s between the noun and verb. For the NV pair, another +/− sign was simultaneously presented for 2.7 s. This sign indicated the correctness of the NV pairing, either matched (+) or mismatched (−) with the SV pairs in the sentence structure. Note that there were two SV pairs in each Sentence (see

Table 1). In the NV pairs, the nouns were presented always without a suffix for the accusative case, and the verbs were presented always with a third-person singular suffix in the simple past tense.

All participants in the present study successfully completed the G1-G3 conditions in the previous study [1]. Given that the participants were familiar with the Kazakh lexical items to be used in the present study, the Lexical list was not shown during the demo trials under the G4 condition. For most participants (23 out of 31), the first session in the G4 condition was conducted on the same day that they completed the G3 condition.

During the scans, the participants wore MRI-compatible video goggles (resolution = 800 × 600 pixels, framerate = 60 fps; VisuaStim Digital, Resonance Technology Inc., Northridge, CA). A small red cross was always shown at the center of the goggle screen. The participants were instructed to look at the small red cross as much as possible during the tasks. The Presentation software package (Neurobehavioral Systems, Albany, CA) supported and controlled the presentation of the stimuli and the collection of the behavioral data [accuracy and response times (RTs)].

***Tasks***

In each task trial (Fig. 2b), a Lexical list was presented as explained above, and then a Sentence was presented after a pause of 0.75 s. We tested the grammaticality task ("GR task"), in which a Sentence using all of the five words in the Lexical list was presented. The symbols of a blue "+" and yellow "–" were then presented. The participants judged whether the Sentence was grammatical (+) or ungrammatical (−), and responded within 3.0 s by pressing one of the two colored buttons (blue or yellow) on a response pad ("GR response"). After a pause of 0.75 s, we tested the subject-verb task ("SV task"), in which an NV pair was presented. The symbols of a green "+" and red "–" were then presented. The participants

judged whether the NV pair was matched (+) or mismatched (−) with the SV pairs in the sentence structure, and responded within 3.0 s by pressing one of the two colored buttons (green or red) ("SV response"). We measured RTs from the onset of the GR or the SV response.

The demo and task blocks under the G4 condition were given over one to three days, with possible intervals of 12 ± 15 days (mean ± SD; range: 1-63 days). At the beginning of each day, the participants reviewed their knowledge of the G1-G3 conditions outside of the scanner, until the participants performed accurately for each condition (at least six out of the eight task trials for both the GR and SV tasks). A one-day experiment (equal to or less than 12 scanning runs) took less than 2.5 hours, including instructions (see the *Introduction*) along with one or two breaks outside of the scanner.

As tested in our previous experiment [1], three of the "Words" trials in a scanning run were followed by a block of eight G4 trials, and then by two Words trials (i.e., 13 task trials in total). Each Words trial began with a visual cue of "W," followed by the presentation of a Lexical list of five words [two verbs, two nouns, and *adamdï* (or *adam*); the order was maintained throughout all trials]. In the Words trials, the Lexical list was basically the same as in the G4 trials, except that those words could have suffixal changes. A set of five words ("Lexicons") was then presented auditorily to the participants. The Lexicons consisted of the same five words as the Lexical list in the same order, but one to four of them had a different suffix. The duration of the Lexicons phase was adjusted to 4.35 s. The symbols of a blue "1," yellow "2," green "3," and red "4" were then presented. The participants judged the number of words that had a different suffix, and responded within 3.0 s by pressing one of the four colored buttons of a response pad ("Words response").

***fMRI data analyses***

The acquisition timing of each axial slice of the MRI scans (see the main text) was corrected using the middle slice (the 15th slice chronologically) as a reference for the functional images. We spatially realigned each volume to the first volume of consecutive runs, and a mean volume was then calculated. We set the threshold for head movement during a single run within a displacement of 2 mm in any of the three directions, and within a rotation of 1.4° around any of the three axes. These thresholds were empirically determined from our previous studies [1,2]. If a run included one or more images over this threshold, we replaced the outlying image with an interpolated image. This interpolated image was the average of the chronologically former and latter ones of the outlying image. We then spatially realigned each volume to the first volume of consecutive runs again. Due to excessive head movement, even after this realignment procedure was conducted, one or two runs were excluded from the analyses for three participants. The realigned data were resliced every 3 mm using seventh-degree B-spline interpolation. Each participant's structural image was matched with the mean functional image generated during realignment. The resultant structural image was spatially normalized to the standard brain space as defined by the Montreal Neurological Institute (MNI). This was accomplished by using the extended version of the unified segmentation algorithm with light regularization. This is a generative model that combines tissue segmentation, bias correction, and spatial normalization in a single model [3]. The resultant deformation field was applied to each realigned functional image to be spatially normalized with non-linear transformations. All normalized functional images were then "smoothed" by using an isotropic Gaussian kernel of 9 mm full-width at half maximum (FWHM).

In the first-level analysis (i.e., the fixed-effects analysis within a participant), each participant's hemodynamic responses were estimated for the following events: Lexical list, Sentence, GR response, NV pair, and SV response (see Fig. 2b), together with Lexicons and

Words response. The events of Sentence, GR response, NV pair, and SV response were separated for each of the OS, OO, SO, and SS conditions. Each event was estimated using the "boxcar" function [taking a constant value during the event period (0 otherwise)], and then that function was overlaid with a hemodynamic response function. Low-frequency noise was removed by a high-pass filtering at 1/128 Hz. To minimize the effects of head movement, the six realignment parameters obtained from preprocessing were included as nuisance factors in a general linear model. The estimated responses for each event were then generated using the general linear model for each participant.

In the second-level analysis (i.e., the random-effects analysis for a group), the above estimated responses were used for the inter-subject comparison. We adopted whole-brain analyses, in which we performed the following tests with three nuisance factors (age, gender, and laterality quotient): one-way repeated measures analyses of covariance (rANCOVAs) within each group (Figs. 4a, 4b, 4d, and 4e), unpaired *t*-tests for direct group comparisons (Fig. 4c), rANCOVAs for all participants (Supplementary Figs. S3a and S3b), and two-way [groups × quarters] rANCOVAs (Supplementary Figs. S3c and S3d). For the anatomical identification of the activated regions, we used the Anatomical Automatic Labeling (AAL) method (http://www.gin.cnrs.fr/AAL2/) [4] and the cortical data with the region names as provided by Neuromorphometrics Inc. (http://Neuromorphometrics.com/) under academic subscription. In addition to the whole-brain analyses, we adopted analyses for each region of interest (ROI) by using the MarsBaR-toolbox (http://marsbar.sourceforge.net/).

# References

1 Umejima, K., Flynn, S. & Sakai, K. L. Enhanced activations in syntax-related regions for multilinguals while acquiring a new language. *Sci. Rep.* **11**, 7296, doi:10.1038/s41598-021-86710-4 (2021).

2 Kinno, R., Kawamura, M., Shioda, S. & Sakai, K. L. Neural correlates of noncanonical syntactic processing revealed by a picture-sentence matching task. *Hum. Brain Mapp.* **29**, 1015-1027, doi:10.1002/hbm.20441 (2008).

3 Ashburner, J. & Friston, K. J. Unified segmentation. *Neuroimage* **26**, 839-851, doi:10.1016/j.neuroimage.2005.02.018 (2005).

4 Tzourio-Mazoyer, N. *et al.* Automated anatomical labeling of activations in SPM using a macroscopic anatomical parcellation of the MNI MRI single-subject brain. *Neuroimage* **15**, 273-289, doi:10.1006/nimg.2001.0978 (2002).
